# Integrative analysis of super enhancer SNPs for type 2 diabetes

**DOI:** 10.1371/journal.pone.0192105

**Published:** 2018-01-31

**Authors:** Weiping Sun, Sihong Yao, Jielong Tang, Shuai Liu, Juan Chen, Daqing Deng, Chunping Zeng

**Affiliations:** 1 Department of Geriatrics, the First People's Hospital of Xiangtan City, Xiangtan, PR, China; 2 Department of Clinical Medicine, Jishou University School of Medicine, Jishou, PR, China; 3 Department of Endocrinology, the Fifth Affiliated Hospital of Guangzhou Medical University, Guangzhou, PR, China; University of Utah, UNITED STATES

## Abstract

Clinical studies in type 2 diabetes (T2D) primarily focused on the single nucleotide polymorphisms (SNPs) located in protein-coding regions. Recently, the SNPs located in noncoding regions have also been recognized to play an important role in disease susceptibility. The super enhancer is a cluster of transcriptional enhancers located in noncoding regions. It plays a critical role in cell-type specific gene expression. However, the exact mechanism of the super enhancer SNPs for T2D remains unclear. In this study, we integrated genome-wide association studies (GWASs) and T2D cell/tissue-specific histone modification ChIP-seq data to identify T2D-associated SNPs in super enhancer, followed by comprehensive bioinformatics analyses to further explore the functional importance of these SNPs. We identified several interesting T2D super enhancer SNPs. Interesting, most of them were clustered within the same or neighboring super enhancers. A number of SNPs are involved in chromatin interactive regulation and/or potentially influence the binding affinity of transcription factors. Gene Ontology (GO) analysis showed a significant enrichment in several well-known signaling pathways and regulatory process, *e*.*g*. WNT signaling pathway, which plays a key role in T2D metabolism. Our results highlighted the potential functional importance of T2D super enhancer SNPs, which may yield novel insights into the pathogenesis of T2D.

## Introduction

Type 2 diabetes (T2D) is a long-term metabolic disorder which is characterized by hyperglycemia and the aberrant metabolism of fat and protein due to the deficient action of insulin [1]. Over the past two decades, the increasing prevalence of T2D has become a serious public health burden with over 4.9 million deaths in 2014 throughout the world [2], and it is estimated that 439 million people would suffer T2D by 2030 [3]. T2D has become one of the leading causes of death, disability, and the rising cost of health care. Compelling evidence showed that the risk of T2D is strongly influenced by genetic factors [4]. Recently, the successive waves of genome-wide association studies (GWASs) have inferred more than 80 robust T2D single nucleotide polymorphisms (SNPs), but in aggregate, these SNPs explained only a small fraction of the T2D heritability [5].

Previous studies have shown that SNPs in protein-coding region are associated with hundreds of diseases by affecting the expression of their target genes [6]. However, over 90% of GWAS-identified variants lie within noncoding regions and the mechanisms of how these SNPs contribute to disease susceptibility remain unclear [7]. Super enhancer, a cluster of transcriptional enhancers, overlapped with noncoding regions (*e*.*g*. DNA methylation valleys and locus control regions) which plays an important role in cell-type specific gene expression [8]. A recent study showed that the SNPs associated with specific diseases are particularly enriched in the super enhancers of disease-relevant cell/tissue types [9]. For example, the SNPs associated with Alzheimer’s disease are enriched in super enhancers of human brain tissue, and super enhancers in B and T cells are enriched for SNPs which are associated with rheumatoid arthritis [9]. However, the role of cell/tissue-type specific super enhancer SNPs for T2D is still unknown.

In this study, we integrated T2D GWAS meta-analysis dataset with T2D cell/tissue-specific histone modification ChIP-seq data to identify T2D super enhancer SNPs, followed by comprehensive bioinformatics analyses to further elucidate the potential functional importance of these SNPs in the pathogenesis of T2D. Our results may yield novel insights into the genetic basis of T2D.

## Materials and methods

### T2D GWAS datasets

We retrieved 792 potential T2D SNPs at GWAS significance threshold (p-value < 5×E-8) from Diabetes Genetics Replication and Meta-analysis (DIAGRAM) Consortium (http://diagram-consortium.org/downloads.html). To our knowledge, it is the standard and the largest T2D meta-analysis study which conducted in 34,840 cases and 114,981 controls of European descent. Next, we used proxy SNAP (https://www.broadinstitute.org/mpg/snap/ldsearch.php) to identify SNPs in LD with retrieved T2D SNPs. The search was based on genotype data from the 1000 Genomes Project with the Utah residents with European ancestry (CEU) population panel. The inclusion criteria for LD SNPs were set as a distance limit of 500 KB from the query SNP with r^2^ > 0.8 from pairwise LD calculations. A total of 1,086 potential functional T2D SNPs was identified.

### Histone modification datasets

We selected T2D-relevant cell/tissues based on their crucial role in pathogenesis of T2D through comprehensive, systematic literature searches in PubMed. In total, we identified 27 T2D-relevant cell/tissue types, such as adipose tissue [10–13], aorta [14–16], bladder [17–19], liver [20–22], T cell [13, 23–25], pancreas [26–29], thymus [30], brain [31–34], osteoblasts [35], skeletal muscle and myoblast [36–38]. The human histone modification WIG/BIGWIG files (H3K27ac) in 27 T2D-relevant cell/tissue types were downloaded from Gene Expression Omnibus (GEO) database (https://www.ncbi.nlm.nih.gov/geo/). A detailed list of all T2D-relevant cell/tissue types and sample information are included in **S1 Table**.

### Identification of T2D super enhancer and T2D super enhancer SNPs

Histone modification WIG/BIGWIG files were mapped to the human reference genome (hg19) using Bowtie v1.1.2 (http://bowtie-bio.sourceforge.net/index.shtml). MACS v1.4.1 was used to identify enhancer regions [39], it applies a dynamic parameter, λ_local_, to calculate the p-value of each candidate peak and removes potential false positives due to local biases. The significant threshold was defined as Bonferroni adjust p-value < 1×E-9. Super-enhancers were separated from enhancers using the ROSE algorithm [40]. A detailed description of the super enhancer identification process was illustrated in the previously published pipeline [9]. We identified a total of 18,422 super enhancers in 27 T2D-relevant cell/tissue types (**S2 Table**) and mapped 1,086 potential functional T2D SNPs to these super enhancers. Finally, we achieved 286 T2D super enhancer SNPs (**S3 Table**).

### Functional prediction of T2D super enhancer SNPs

Characterization of the regulatory features of T2D super enhancer SNPs was carried out through the bioinformatics tool rVarBase [41]. rVarBase utilizes experimentally supported regulatory elements from ENCODE and other data resources to make relevant annotation, it provides reliable regulatory feature for human variants. rVarBase annotates regulatory feature of variants from chromatin state of the queried variant surrounding region, regulatory elements that overlapped with the queried variant, and potential target genes of queried variant. To further validate the potential functional consequence of T2D super enhancer SNPs, we applied HaploReg v4.1 [42], a comprehensive resource which utilizes data from Roadmap Epigenomics, ENCODE and Genotype-Tissue Expression Project (GTEx) *etc*., to explore the effect of T2D super enhancer SNPs on gene expression (eQTLs) and regulatory motif alterations within sets of genetically linked T2D super enhancer SNPs. Furthermore, we performed transcription factor enrichment analysis using SNP2TFBS (http://ccg.vital-it.ch/snp2tfbs/) tool which selects and visualizes user defined variants that affect single or multiple transcription factors.

### Long-range interaction analysis of T2D super enhancer SNPs

GWAS3D (http://jjwanglab.org/gwas3d) systematically calculates the probability of GWAS-associated SNPs which affect regulatory pathways and underlie disease associations by integrating functional genomics, chromatin state, sequence motif, and conservation information [43]. Moreover, the results can be interpreted by comprehensive visualization. In this study, we used GWAS3D to identify the significant T2D super enhancer SNPs which have a long-range interaction signal with their distal regulatory elements using the system default parameters: Fisher’s combined p-value < 1×E-5, r^2^ = 0.8, CEU population panel (HapMap phase I+II+III), binding site p-value = 0.01, size of 30 variants, and 3 interaction size.

### Gene ontology (GO) analysis

To find out the functional enrichment (*e*.*g*. biological processes) of T2D super enhancer SNPs, we assigned each T2D super enhancer SNPs to their nearest genes based on the human genome assembly GRCH37 (hg19) from Genome Reference Consortium. We carried out GO analyses using these T2D super enhancer SNPs target genes through GOEAST toolkit (http://omicslab.genetics.ac.cn/GOEAST/). The p-values were calculated by hypergeometric tests and adjusted for multiple comparisons using stringent Yekutieli (FDR under dependency) method [44]. The statistical significant threshold was defined as Yekutieli adjust p-value < 0.05.

### Protein–protein interaction networks

STRING v10.0 (http://string-db.org/) is a database of known and predicted protein interaction, including physical and function association. It quantitatively integrates interaction data derived from genomic context, high-throughput experiments, co-expression and previous knowledge [45]. The database currently contains 9.6 million proteins from 2031 organisms constructed more than 184 million interactions. In order to explore the functional partnership and interaction of the identified T2D super enhancer SNP target genes (**S3 Table**), we construct gene (protein) interaction networks via the STRING v10.0 database with default settings (observed interaction, 70; expected interaction, 8.83; Benjamini adjust p-value < 1×E-10; proteins, 76).

## Results

### Identification of T2D super enhancer SNPs

In this study, we first retrieved 792 potential T2D SNPs at GWAS significance threshold (p-value < 5×E-8) from the largest T2D meta-analysis study DIAGRAM Consortium. By using LD information from the 1000 Genomes Project with CEU population panel, we inferred 1,086 potential functional T2D SNPs. Then we took advantage of the public available human histone modification ChIP-seq datasets from GEO database to construct a comprehensive list of super enhancer in T2D-relevant cell/tissue types. In total, we examined and confirmed 18,422 super enhancer regions in 27 human cell/tissue types (**S2 Table**). Then 1,086 potential functional T2D SNPs were mapped to the identified T2D cell/tissue-specific super enhancer. Finally, we obtained a total of 286 potential functional T2D super enhancer SNPs (**S3 Table**).

**Fig 1:**
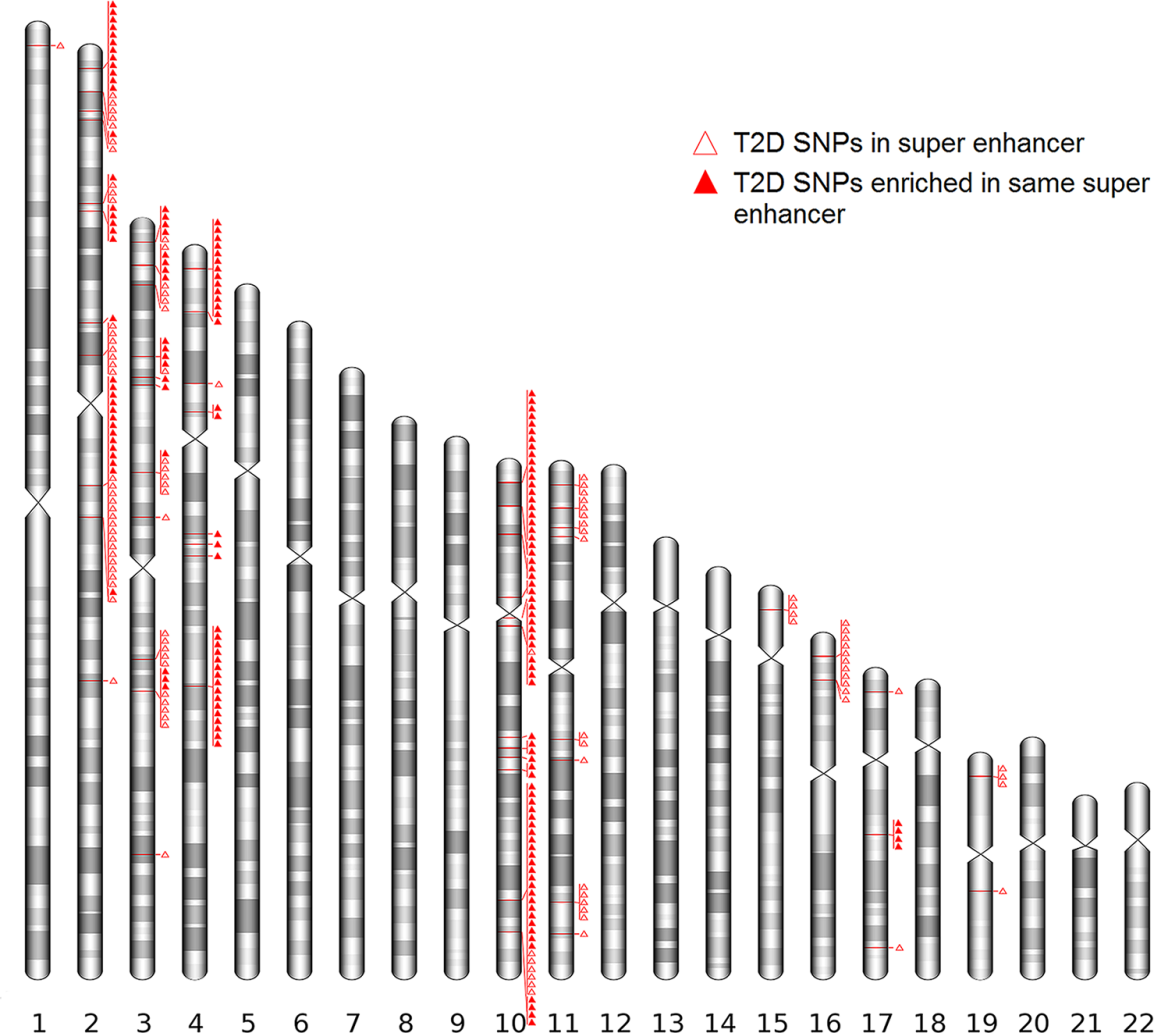
Distribution of T2D super enhancer SNPs in human genome. T2D super enhancer SNPs were annotated to the human genome assembly GRCH37 (hg19). The enrichment of T2D super enhancer SNPs was observed on chromosome 2p21 (*THADA*), 3q21.1 (*ADCY5*), 4p16.1 (*WFS1*) and 10q25.2 (*TCF7L2*).

To identify whether T2D super enhancer SNPs enriched in specific cell/tissue types, we characterized the distribution of the T2D super enhancer SNPs in different cell/tissue types and identified a significant SNP enrichment in adipose tissue (161/286), aorta (115/286), pancreas (113/286) and brain (44/286) (**S4 Table**). Notably, 160 SNPs were mapped to super enhancer in a wide variety of cell/tissue types (**S4 Table**). Interestingly, some of these SNPs enriched in the same or neighboring super enhancers, the strong enrichment of T2D super enhancer SNPs on the chromosome 2p21 (*THADA*), 3q21.1 (*ADCY5*), 4p16.1 (*WFS1*) and 10q25.2 (*TCF7L2*) are rather striking (**Fig 1)**. In additional, we checked the LD status among each of the SNPs pairs that mapped to the same or neighboring super enhancers. To our surprise, we identified several SNP pairs with low LD which were mapped to the same super enhancers (**S4 Table & S5 Table**), *e*.*g*. the SNP pair rs11196182 and rs12245680 with low LD (r^2^ = 0.016) were mapped to the same super enhancer in adipose tissue (10q25.2). The full list of SNPs that map to a specific super enhancer and LD between those SNPs is included in **S5 Table**.

### Functional prediction of T2D super enhancer SNPs

To investigate the potential functional impact of the identified T2D super enhancer SNPs, we annotated 286 T2D super enhancer SNPs to various regulatory elements through rVarBase [41]. rVarBase is a database which utilizes experimentally supported regulatory elements from multiple data resources to make relevant functional prediction, it provides reliable regulatory feature for human variants [41]. Finally, we identified 57 regulatory SNPs which were supported by experimental evidences. Of the 57 SNPs identified, 20 SNPs are involved in chromatin interactive regulation, 4 SNPs are involved in regulation of lncRNA expression, and 4 SNPs overlapped with CpG islands (**S6 Table)**. In addition, to validate the potential functional consequence of the T2D super enhancer SNPs, we investigated the effect of T2D super enhancer SNPs on regulatory motifs and gene expression (eQTLs) by using data from ENCODE and GTEx projects through HaploReg v4.1. Finally, we observed that 242 T2D super enhancer SNPs changed transcription factor binding motif, this result highlighted the strong regulatory potential of these T2D super enhancer SNPs (**S7 Table**). Moreover, we identified 186 T2D super enhancer SNPs which were reported eQTL evidences, including 120 T2D super enhancer SNPs showed eQTL evidences in a wide variety of tissues and cell types (**S7 Table**). Importantly, we found that all 57 regulatory SNPs identified by rVarBase were replicated in HaploReg regulatory prediction results (**S6 Table & S7 Table**).

**Fig 2:**
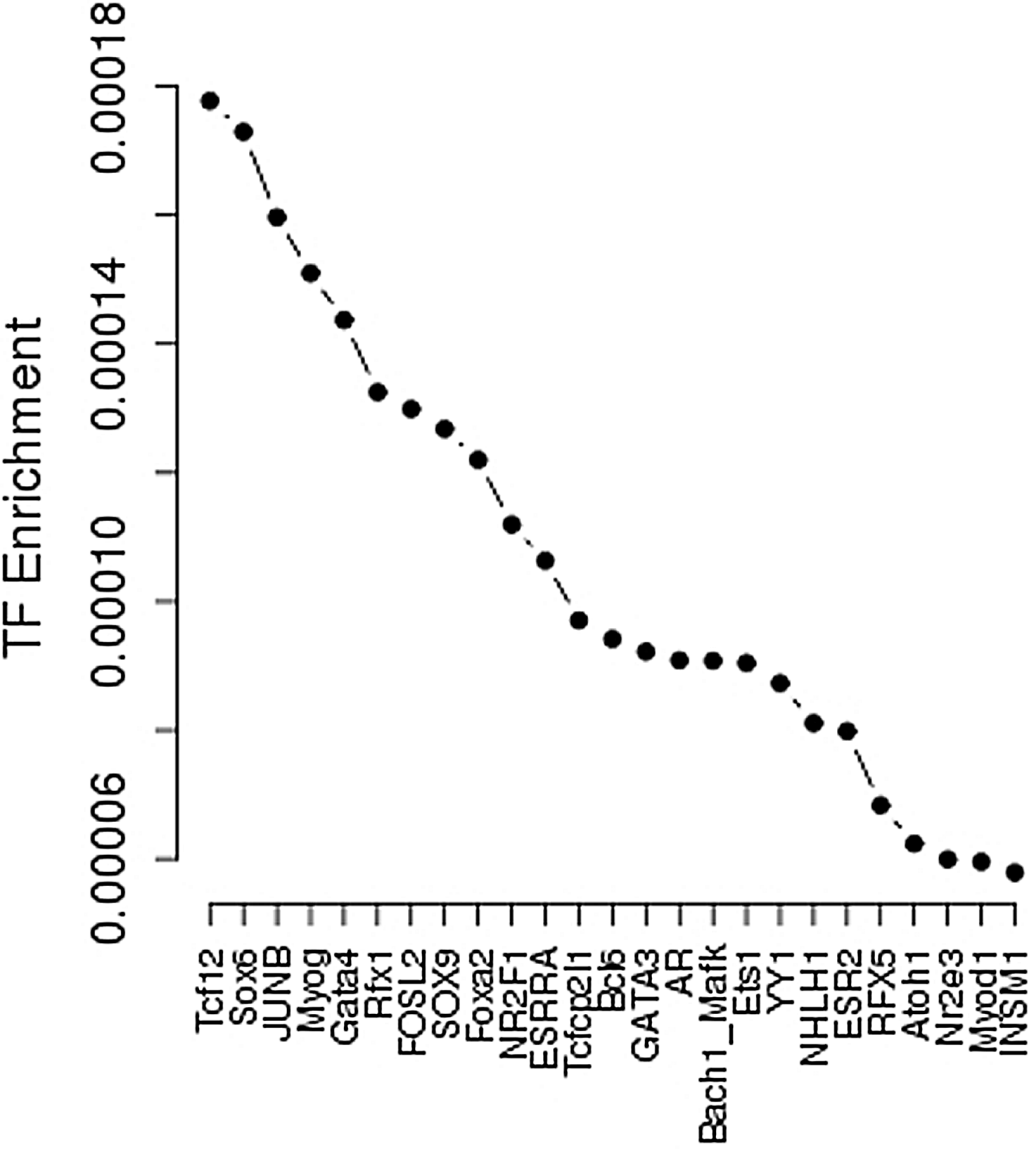
Magnified transcription factors enrichment plot for T2D super enhancer SNPs. Transcription factors are sorted based on their enrichment.

The effect of T2D super enhancer SNPs on the transcription factor binding affinity was further analyzed by SNP2TFBS. The top five enrichment transcription factors are Tcf12, Sox6, JUNB, Myog, and Gata4 (**Fig 2**). Transcription factors Tcf12 is an important component member of WNT signaling and ERK signaling pathway. Diabetogenic factors have been found to influence insulin signaling through activation of the ERK signaling pathway [46]. Sox6 and JUNB are involved in T2D metabolism, T2D-related cell development and differentiation [47, 48]. Gata4 is important transcription factor which is involved in the regulation of T2D development and metabolism [49].

### Long-range interaction of T2D super enhancer SNPs

**Fig 3:**
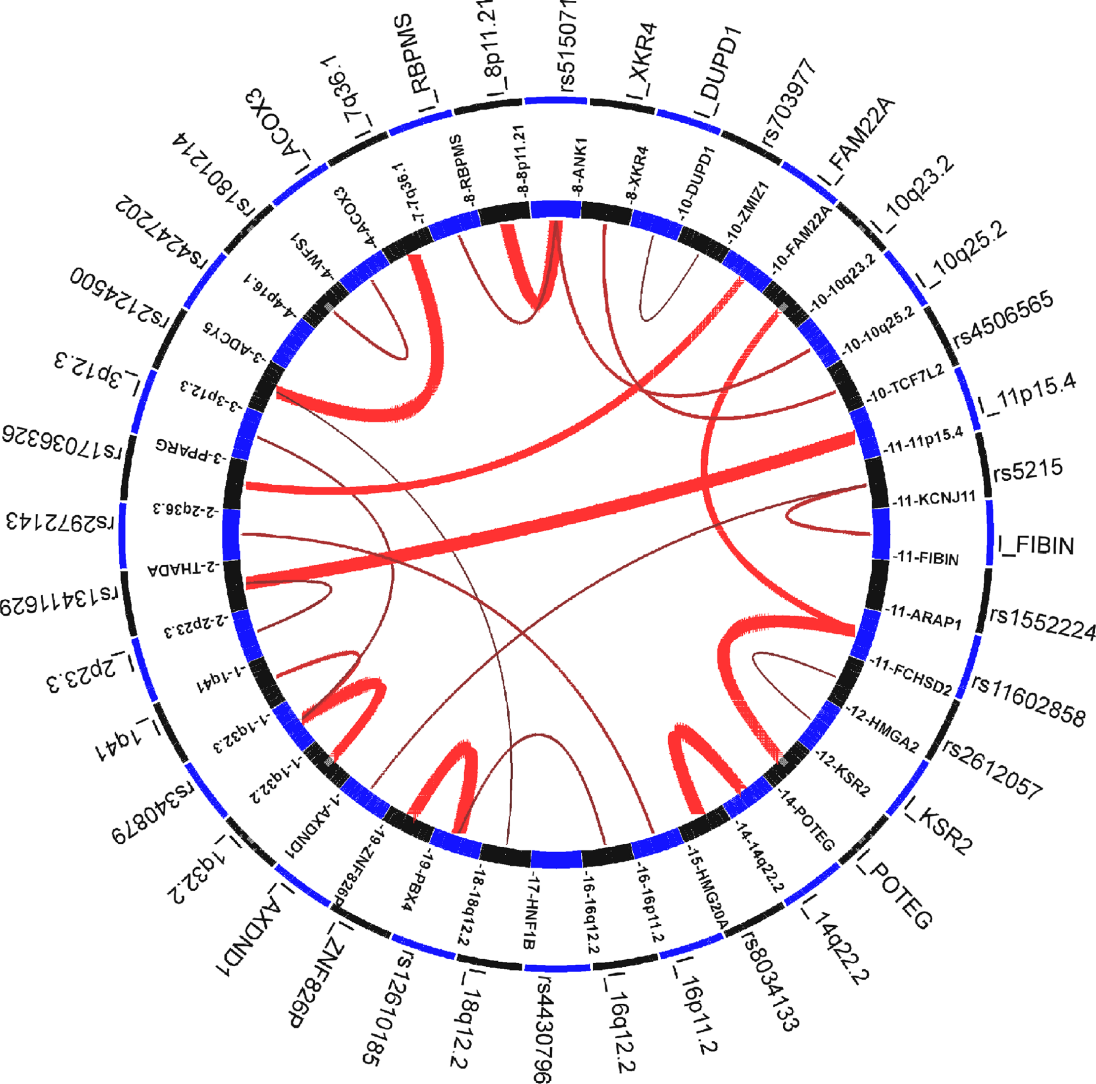
The circle plot of GWAS3D for T2D super enhancer SNPs. The annotation based on all cell line and CEU population. The red line indicated long-range interaction signals, and the intensity of interaction was represented by the width of the line. Interactive elements with significant SNP will start with ‘I_’.

GWAS3D was used to identify T2D super enhancer SNPs which have a long-range interaction signal with their distal regulatory elements. Finally, we detected 82 SNPs (Fisher’s combined p-value < 1×E-5) affecting the long range interaction based on the CEU population and all cell types listed in GWAS3D (**Fig 3, S8 Table**). For example, SNP rs13411629, located in the intronic region of *THADA* on chromosome 2, has a long-range interaction signal with locus 11p15.4. SNP rs2124500, located in the intronic region of *ADCY5* on chromosome 3, has two long-range interaction signals with locus 7q36.1 and 18q12.2. SNP rs1552224, located in the intronic region of ARAP1 on chromosome 11q13.4, showed two long-range interaction signals with locus 10p23.2 and locus near POTEG respectively. Furthermore, GWAS3D also revealed 105 SNPs which may affect promoter activity by changing transcription factor binding site affinity (**S8 Table**), including 76 SNPs have direct effect by GWAS leading SNPs and 29 variants have indirect effect by high LD of GWAS leading SNPs. Interestingly, among the 105 functional SNPs identified by GWAS3D, 96 SNPs were also confirmed by HaploReg regulatory prediction results (**S7 Table & S8 Table**).

### GO analysis of T2D super enhancer SNPs target genes

To systematically investigate whether the identified T2D super enhancer SNPs were specifically associated with T2D, we conduct GO analysis using 26 T2D super enhancer SNPs target genes (**S3 Table**) through GOEAST. We identified 151 significant GO terms with Yekutieli adjust p-value < 0.05 (**S9 Table**). Interestingly, we observed a significant enrichment in signaling pathways and regulation process (**Table 1, S9 Table**), *e*.*g*. WNT signaling pathway and G-protein coupled receptor signaling pathway, which play a key role in the pathogenesis of T2D [50, 51]. Other interesting GO terms, such as positive regulation of lipid metabolic process and negative regulation of inflammatory response, may also have a potential function in the metabolism of T2D.

**Table 1:** The most significant GO terms for T2D super enhancer SNPs target genes.

GOID	Term	log_odds_ratio	p-value
GO:0048008	platelet-derived growth factor receptor signaling pathway	6.72	6.97E-13
GO:0010172	embryonic body morphogenesis	7.70	7.26E-10
GO:0050794	regulation of cellular process	1.14	4.98E-08
GO:0023052	Signaling	1.64	1.10E-07
GO:0044700	single organism signaling	1.64	1.10E-07
GO:0050789	regulation of biological process	1.08	1.90E-07
GO:0007154	cell communication	1.59	2.04E-07
GO:0065007	biological regulation	1.02	3.75E-07
GO:0007166	cell surface receptor signaling pathway	2.27	6.59E-07
GO:0051974	negative regulation of telomerase activity	7.88	9.20E-07
GO:0016055	WNT signaling pathway	3.99	3.00E-06
GO:0198738	cell-cell signaling by WNT	3.99	3.00E-06
GO:0007165	signal transduction	1.57	3.07E-06
GO:0007186	G-protein coupled receptor signaling pathway	3.21	3.19E-06
GO:0051972	regulation of telomerase activity	7.39	3.53E-06

GO enrichment analysis was performed using the GOEAST tool. The p-values were calculated by hypergeometric tests and adjusted for multiple comparisons using stringent Yekutieli (FDR under dependency) adjustment.

### Protein–protein interaction networks

**Fig 4:**
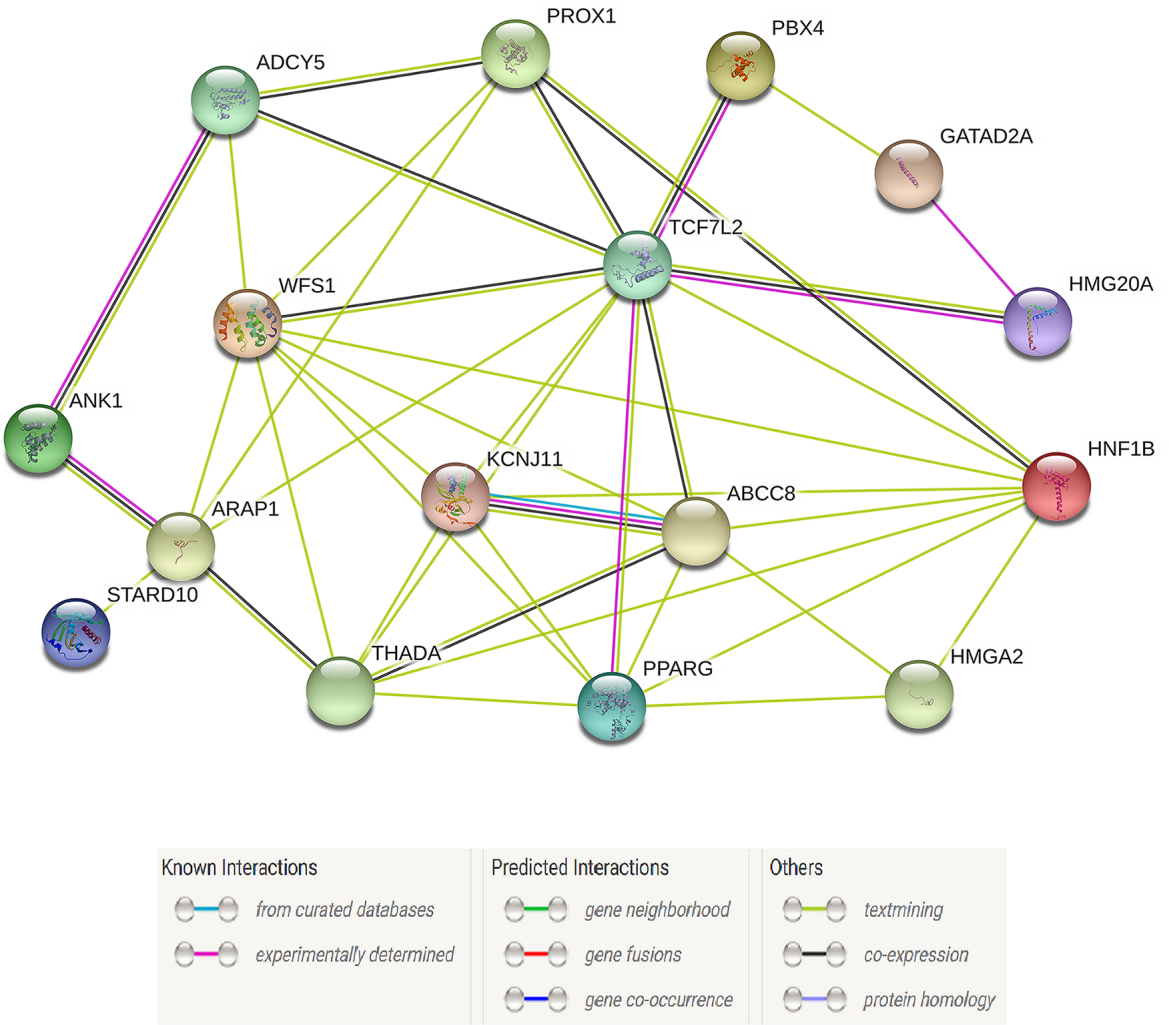
A functional protein-protein interaction network for T2D super enhancer SNPs target genes. Connections are based on co-expression and experimental evidence with a STRING v10.0 summary score above 0.4. Each filled node denotes a gene; edges between nodes indicate protein-protein interactions between protein products of the corresponding genes. Different edge colors represent the types of evidence for the association.

To partially characterize the functional partnership and their interaction networks among the identified T2D super enhancer SNPs target genes, we uploaded the 26 T2D super enhancer SNPs target genes (**S3 Table**) into STRING v10.0 database with default settings (observed interaction, 70; expected interaction, 8.83; Benjamini adjust p-value < 1×E-10; proteins, 76). **Fig 4** revealed a strong association between the topological properties and biological function of T2D target genes. The hub genes with the strong connections were *KCNJ11*, *PPARG*, *ABCC8*, *THADA*, *WFS1*, *TCF7L2*, and *ADCY5*. Interestingly, these genes are involved in the energy reserve metabolic process, glucose homeostasis, and negative regulation of pancreatic β cell apoptotic process, which revealed their potential functional impacts on the pathogenesis of T2D.

## Discussion

In the human genome, there are much more genetic variations located in the noncoding regions when compared with protein-coding regions. Some important functional regulatory elements, such as the super enhancer, have a great impact on cell-type specific gene expression. SNPs located in super enhancer may play an essential role in disease metabolism. In this study, we integrated GWASs data from DIAGRAM and T2D cell/tissue-specific histone modification ChIP-seq data from GEO to identify T2D super enhancer SNPs. To explore the potential functional consequence of these SNPs and their target genes, we characterized their regulatory feature using several different database, performed transcription factor enrichment analysis, long-range interaction analysis, and conduct GO and protein-protein interaction network analysis.

Our results indicate a total of 286 potential functional T2D super enhancer SNPs. Interestingly, some of these SNPs showed a strong enrichment in the T2D genes or novel candidate genes, *e*.*g*. *ADCY5*, *ANK1*, *PPARG*, *TCF7L2*, *THADA*, *WFS1*, and *ZMIZ1*. We identified 18 super enhancer SNPs enriched in T2D-associated gene *ADCY5*, this gene encodes a member of the membrane-bound adenylyl cyclase enzymes which are expressed in heart, brain, and pancreatic β cells, *etc*. Adenylyl cyclases mediate G-protein coupled receptor signaling through the synthesis of the second messenger cAMP, which can modulate glucose-stimulated insulin secretion by several possible mechanisms [52]. Another interesting gene *ANK1*, located on chromosome 8p11.21, contains 6 super enhancer SNPs, this gene encodes a member of the ankyrin family which links the integral membrane proteins to the spectrin-actin cytoskeleton [53]. It is expressed in several human tissues, *e*.*g*. endocrine pancreas, which is relevant to the glucose metabolism [54]. A recent study reported that variants in *ANK1* were significantly associated with the susceptibility to T2D and there exists tissue-specific expression profile of *ANK1* in the human islet, pancreas, skeletal muscle, adipose and liver tissues which are important organs for glucose metabolism [55]. There are 16 super enhancer SNPs enriched in *PPARG* gene. This gene encodes a member of the nuclear receptor family of ligand-activated transcription factors and is predominantly expressed in adipose tissue [56]. Werman *et al*. suggested that *PPARG* is a major regulator of adipogenesis via stimulation by fatty acids, *e*.*g*. insulin enhances the transcriptional activity of the *PPARG* [57]. Importantly, we identified that super enhancer SNPs were highly enriched (88/286) in *TCF7L2* gene. Variants in *TCF7L2* have been associated with T2D in multiple ethnic groups [58–60]. As a transcription factor, *TCF7L2* plays a critical role in WNT signaling pathway and is involved in the development of various cell lineages and tissues [61]. Potential mechanisms of *TCF7L2* SNPs influencing on T2D include its role in adipogenesis, pancreatic islet development, and insulin secretory granule function.[62]. It is also involved in impaired glucose production and tolerance via direct effects on pancreatic β cells [63].

Functional annotation of T2D super enhancer SNPs through rVarBase showed 57 regulatory SNPs, including 20 SNPs which are involved in chromatin interactive regulation, 4 SNPs which are involved in regulation on lncRNA, and 4 SNPs overlapped with CpG islands. Additional functional prediction analysis showed that all these 57 regulatory SNPs were replicated in HaploReg results, which highlighted the strong and reliable regulatory potential of these T2D super enhancer SNPs. Interestingly, 22 out of 54 regulatory SNPs targeted to gene *WFS1*, this gene encodes a transmembrane protein which maintains calcium homeostasis of the endoplasmic reticulum and is ubiquitously expressed in brain, pancreas, heart, and pancreatic β cell lines [64]. *WFS1* is critical for the endoplasmic reticulum stress response in insulin-producing pancreatic β cells which contributes to the risk of T2D [64]. A recent study shows that the SNPs associated with prostate cancer can modulate lncRNA expression. This result indicates that lncRNA may play an essential role in human complex disease. Notably, we identified 4 regulatory SNPs, rs508419 in gene *ANK1*, rs4689393, rs3821941 and rs4688987 in gene *WFS1* which are involved in regulation on lncRNA, SNP rs508419 also targeted to a CpG island. In addition, we indicated that T2D super enhancer SNPs may alter the transcription factor binding affinity of Tcf12, Sox6, JUNB, Myog, and Gata4, *etc*. For example, transcription factors Gata4 is critical for the regulation of T2D development and metabolism [49]. Transcription factors Sox6 and JUNB are involved in T2D metabolism, T2D-related cell development and differentiation [47, 48]. Furthermore, we examined the effect of T2D super enhancer SNPs on expression of their target genes (eQTLs) by using data from GTE projects, 186 T2D super enhancer SNPs showed eQTL evidences, including 120 T2D super enhancer SNPs have numerous reported eQTL evidences in a wide variety of tissues and cell types.

GWAS3D revealed 82 T2D super enhancer SNPs which have long-range interaction signals. As long-range interaction can also be cell/tissue type specific, we performed additional analysis to examine whether long-range interaction of T2D super enhancer SNPs and their distal regulatory elements occurred in the same cell/tissue type. Due to the limited cell/tissue types listed in GWAS3D, we did not find the direct long-range interactions in the same cell/tissue type. However, we discovered 21 T2D super enhancer SNPs in adipose tissue which showed long-range interactions in K562 cell line. This cell line was the first human immortalised myelogenous leukemia line to be established [65]. Interestingly, among the 21 T2D super enhancer SNPs in adipose tissue, 13 SNPs were mapped to *TCF7L2* gene. This gene encodes a WNT transcription factor which has been linked to a higher risk of developing T2D [58–60, 66]. Previous studies have also indicated that WNT signaling pathway is the most commonly dysregulated process in myelogenous leukemia. Notably, *TCF7L2* plays a crucial role in maintaining the proliferation and viability of myeloid leukaemia cells, and overexpression of *TCF7L2* is associated with poor clinical outcome [67]. *TCF7L2* maybe a valid target in myelogenous leukemia therapy. In addition, according to a recently published study, the myelogenous leukemia treatment drug Gleevec has been stumbled on possible use for curing T2D [68]. The researchers discovered that Gleevec blocks CDK5-mediated PPARγ phosphorylation, and thus lowers the level insulin resistance and reduces the risk of hyperglycemia and obesity. They also observed that Gleevec reduce lipogenic and gluconeogenic gene expression in liver and improved inflammation in adipose tissues. Although Gleevec is a new hope for both myelogenous leukemia and T2D cure, the exact mechanisms and their functional relationships remain unclear.

GO analysis of T2D super enhancer SNPs target genes not only confirmed well known WNT signaling pathway and G-protein coupled receptor signaling pathway, but also identified many other mechanisms which may likely contribute to T2D pathogenesis, such as positive regulation of lipid metabolic process and negative regulation of inflammatory response. Protein–protein interaction network analysis identified several interacted hub genes (protein), *e*.*g*. *KCNJ11*, *PPARG*, *ABCC8*, *THADA*, *WFS1*, *TCF7L2*, and *ADCY5*. Most of them have been reported to be associated with the pathogenesis of T2D or T2D-related metabolic pathways, *e*.*g*. gene *WFS1* is involved in the energy reserve metabolic process, glucose homeostasis and negative regulation of pancreatic β cells apoptotic process [69].

In the current study, we performed comprehensive bioinformatics analyses to demonstrate the functional importance of the T2D super enhancer SNPs. However, the databases used in these bioinformatics analyses are specialized, *e*.*g*. we only focus on SNPs from the 1000 genomes project with MAF > 0.001. We admit that a number of known variants satisfying the MAF criterion were missed in the functional prediction analysis. In additional, the results of functional annotation exclusively depend on computationally predicted regulation features, therefore the model and algorithm selection is critical for this kind of analysis. For example, the reliability of the transcription factor binding site predicted by SNP2TFBS is a function of the accuracy of the PWM model which is less accurate than recently proposed approaches [70]. Further, genomic sequence with higher affinity to a transcription factor is often insufficient for in vivo binding. A number of other factors may impact on binding such as chromatin accessibility [71]. Therefore, the functional importance of these candidate T2D super enhancer SNPs may be overshadowed, further experiment validation should be conduct to confirm the functional mechanism of these potential T2D super enhancer SNPs.

In conclusion, by integrating T2D GWAS and histone modification ChIP-seq data, we identified hundreds of T2D super enhancer SNPs. Comprehensive bioinformatics analyses of T2D super enhancer SNPs highlighted their potential functional importance in the regulation of T2D pathogenesis, which may yield novel insights into the genetic basis of T2D.

## Supporting information

S1 Table A detailed list of T2D-relevant cell & tissue types. (XLSX)

S2 Table Super enhancers in T2D-relevant cell & tissue types. (XLSX)

S3 Table The potential functional T2D super enhancer SNPs. (XLSX)

S4 Table The distribution of T2D super enhancer SNPs in different cell & tissue types. (XLSX)

S5 Table List of SNPs that map to the same super enhancer and LD between those SNPs. (XLSX)

S6 Table Functional prediction of T2D super enhancer SNPs by rVarBase. (XLSX)

S7 Table Functional prediction of T2D super enhancer SNPs by HaploReg. (XLSX)

S8 Table Long-range interaction analysis of T2D super enhancer SNPs. (XLSX)

S9 Table GO analysis of T2D super enhancer SNPs target genes. (XLSX)
